# Enhanced IoT Spectrum Utilization: Integrating Geospatial and Environmental Data for Advanced Mid-Band Spectrum Sharing

**DOI:** 10.3390/s24185885

**Published:** 2024-09-11

**Authors:** Colin Brown, Bo Rong

**Affiliations:** Communications Research Centre, Ottawa, ON K2H 8S2, Canada; colin.brown@ised-isde.gc.ca

**Keywords:** spectrum sharing, spectrum database, secondary access, Internet of Things

## Abstract

The anticipated surge of Internet of Things (IoT) devices is expected to intensify the demand for mid-band spectrum resources, posing challenges to traditional spectrum sharing methods. This paper addresses the limitations of static database-assisted spectrum management frameworks and proposes a novel approach integrating high-resolution geospatial and real-time environmental data. Leveraging these inputs, the proposed framework enhances spectrum allocation accuracy, and mitigates interference more effectively, thereby increasing the access opportunities for IoT deployments. A detailed example scenario illustrates the efficacy of the proposed approach, demonstrating significant gains in spectrum sharing efficiency. It shows gains in the number of new entrants accessing the spectrum, ranging from 77% to 140%. These gains occur when moving to less conservative interference conditions and including more complex geospatial information in the propagation environment. These findings underscore the critical role of advanced spectrum sharing techniques in optimizing spectrum utilization for future IoT networks.

## 1. Introduction

As the landscape of the Internet of Things (IoT) continues to expand at an unprecedented pace, the scarcity of mid-band spectrum resources has emerged as a significant bottleneck [1]. This scarcity necessitates a re-evaluation of traditional spectrum sharing methods. Considering the projections for the number of IoT devices are estimated into the billions of devices [2], effective management of spectrum access and preventing harmful radio interference becomes a primary concern for regulatory authorities.

From a spectrum sharing perspective, regulatory authorities employ various mechanisms to coordinate spectrum users and manage harmful radio interference based on frequency, distance, or time. Of particular interest are scenarios where the spectrum is shared between dissimilar services operating in the same geographical area. Such new sharing paradigms are becoming increasingly important in the context of modern IoT applications. One such innovation is the sharing of frameworks supporting database-assisted dynamic spectrum access (DSA).

Conventional mid-band spectrum sharing frameworks, such as the Licensed Shared Access (LSA) in the European Union (EU) [3] and the Spectrum Access System (SAS) for the Citizens Broadband Radio Service (CBRS) in the United States (US) [4,5,6], were primarily designed around static database systems that record incumbent usage but are now challenged by the dynamic nature of modern IoT applications with requirements for real-time spectrum access.

These sharing frameworks, while pioneering in the early stages of spectrum sharing, are inherently limited by their reliance on static and historical data. Such data constraints severely hinder their capability to make proactive, real-time decisions that are essential in the context of IoT, where devices frequently operate in a dynamic spectrum environment. Furthermore, the limitations of database-assisted systems are becoming increasingly apparent, as they lack the flexibility to adapt to the rapid changes in spectrum demand and do not incorporate local geospatial and environmental data [7]. These limitations lead to suboptimal spectrum allocation and inadequate interference management, directly impacting the efficiency and reliability of IoT operations.

Spectrum sharing is essential in our study due to the anticipated surge in the number of IoT devices, which is expected to intensify the demand for mid-band spectrum resources. Traditional spectrum management frameworks are increasingly inadequate in addressing the needs of these devices, which operate in highly dynamic environments and require real-time spectrum access. Our study addresses this gap by proposing a novel spectrum sharing framework that leverages real-time data and high-resolution geospatial information.

This research examines how integrating high-resolution geospatial and real-time environmental data can enhance spectrum sharing efficiency for IoT applications in mid-band spectrum. We hypothesize that incorporating such data and advanced propagation models will significantly improve spectrum allocation accuracy and interference mitigation. The study aims to develop and evaluate a novel spectrum sharing framework, quantify efficiency improvements, and provide recommendations for evolving spectrum management practices.

Recognizing these limitations, this paper proposes a more robust, data-driven approach to spectrum sharing. This new approach integrates not only the traditional databases but also leverages high-resolution geospatial information and real-time environmental sensing data. These data inputs allow the spectrum management framework to identify demand hotspots and predict spectrum usage patterns with greater accuracy. Moreover, incorporating terrain and clutter models, along with environmental data, the proposed framework enhances the predictive capabilities of spectrum management systems, facilitating more precise and efficient spectrum distribution.

By addressing the shortcomings of existing spectrum sharing frameworks, our approach aims to optimize spectrum utilization, reduce interference, and improve the overall reliability and efficiency of IoT networks. The integration of advanced data sources ensures that spectrum management is adaptive to real-time conditions, thereby supporting the scalability and dynamic requirements of future IoT applications. This research underscores the critical role of advanced spectrum sharing techniques in enabling the continued growth and functionality of IoT ecosystems.

## 2. Related Work

Data-driven spectrum management, based on dynamic spectrum access (DSA) and cognitive radio networks (CRN), aims to optimize underutilized spectrum resources. Recent studies have explored advanced sharing approaches, including adaptive methods [8], hybrid systems [9], and prioritized service schemes [10,11]. The integration of geospatial and environmental data in spectrum management is emerging [12,13], though IoT-specific applications remain underexplored.

The evolution towards data-driven spectrum management with an emphasis on sharing is increasingly recognized as a key innovation to unlock otherwise underused or unused spectrum. Previous research has demonstrated the potential of leveraging data analytics for spectrum insights. For instance, in [14], the authors presented a smart spectrum concept using a spectrum database to store local radio environment information and so optimize the spectrum use for sharing with a focus on autonomous vehicles [15]. Similarly, in [16], the contribution centred on embedding spectrum opportunity data—i.e., opportunities to share in time, frequency, and space—into current spectrum management practices rather than performing a deep analysis on the interference environment.

From an IoT perspective, spectrum sharing has long been noted as a promising solution to accommodate diverse services and applications. For example, the work in [17] provided a concise overview of the various IoT technologies deployed in shared environments; however, database-assisted systems were outside the scope of the survey. This finding contrasts with the work published in [18], where the authors examined a spectrum sharing framework for IoT focused on supporting a network of sensors to increase sharing in TV white space (TVWS) spectrum. Another related contribution examined sharing between 5G and satellite services [19]. In this case, the authors focused on the algorithmic changes to manage interference rather than incorporating diverse geospatial data into a sharing framework.

Moreover, the study in [20] proposed an adaptive spectrum access method in database-driven cognitive radio networks, focusing on maximizing throughput by combining local sensing with database information. Research in [21] developed an integration framework for hybrid spectrum access systems that combines static database information and dynamic sensing results, addressing the challenge of integrating these data sources. The authors in [22] introduced a double-phase dynamic spectrum allocation scheme to support prioritized services, such as real-time multimedia applications, in database-assisted environments. Finally, the work in [23] explored a utility-based distributed learning approach for dynamic spectrum access with a geolocation database, enhancing decision-making in spectrum trading.

Other research on sharing and spectrum management has focused either on the combination of mathematical models with diverse datasets to inform spectrum policy decisions [24] or leveraging big data analytics and sensor data to derive insights on managing the radio access network (RAN) for cognitive systems [25]. In both cases, however, spectrum sharing to enable IoT was not within scope.

From a regulatory perspective, the concept of data-driven methodologies in spectrum sharing aligns with regulatory trends that leverage modern technologies to enhance decision-making processes. For example, the Federal Communications Commission (FCC) in the United States and the Office of Communications (Ofcom) in the United Kingdom have both emphasized the importance of better data in spectrum management and future spectrum sharing initiatives [26,27].

Lastly, previous contributions by the authors in this domain have focused on two main themes. In [28], the authors introduced the concept of evolving database-assisted systems by modifying the interference protection criteria and leveraging diverse datasets such as climate information to improve sharing. In contrast, in [29], the research focused on sharing between broadband and satellite systems with limited analysis of the different propagation models and geospatial data being used.

## 3. Our Contribution

The paper builds upon the work presented in [29] and makes three specific contributions. First, a novel spectrum sharing framework is presented that incorporates geospatial and environmental data to derive data-driven insights that meets the complex requirements of modern IoT systems. Second, an example scenario is presented to illustrate how the applied framework can increase the number of new IoT spectrum users in a mid-band sharing scenario. Lastly, the paper presents a deep-dive analysis on the complex interaction between the density of IoT users, the aggregate interference in the environment and the implications of using different models and datasets in the analysis.

The subsequent sections of this paper detail the limitations of current database-assisted spectrum management systems and highlight their inflexibility in handling the unique demands of IoT spectrum sharing. Next, a novel spectrum sharing framework is presented for modern IoT systems. An example scenario is then introduced to illustrate the application of this framework in a mid-band spectrum sharing scenario. This scenario is followed by simulation results and analysis. The paper concludes with a discussion on the implications of these findings for future spectrum management strategies.

## 4. Database-Assisted Sharing

Many regulatory bodies, in tandem with industry partners, have implemented spectrum sharing models that coordinate and manage spectrum access using a database. These frameworks vary in how they authorize access to the spectrum, with some bands allocating exclusive licences while others accommodating licence-exempt usage, each with their own set of rules for shared usage. The US, for example, employs the CBRS model operating within the 3550–3700 MHz spectrum. This model incorporates a geolocation database within an SAS to facilitate spectrum distribution among primary users, secondary licensees, and licence-exempt general authorized access (GAA) users [6]. Depending on the implementation and the specific administrator of the database, these systems can incorporate various degrees of terrain mapping and above-ground clutter to refine spectrum availability models and mitigate interference. Such data inform the decisions around spectrum allocation, significantly enhancing the accuracy and capacity in planning densely populated IoT environments [30].

The limitations of spectrum management practices when assisted by static databases become evident when compared to dynamic database sharing. Static databases lack the capability to adapt to real-time changes in the environment and spectrum usage, leading to suboptimal spectrum utilization and increased interference. This limitation is particularly important for near real-time IoT applications. Dynamic databases, on the other hand, can leverage real-time data to make more accurate and efficient spectrum allocation decisions.

**Figure 1:**
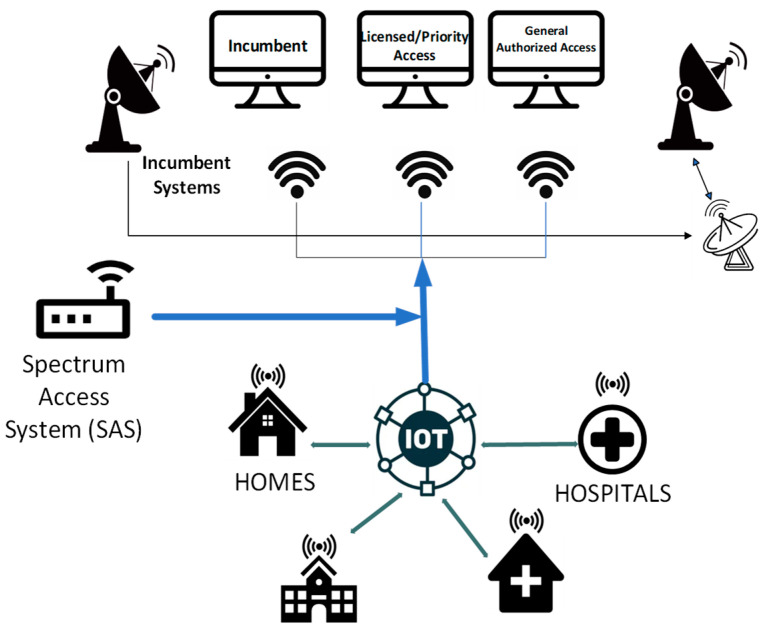
Citizens Broadband Radio Service.

Figure 1 shows a visual representation of a CBRS system including the three-tier nature of the model and the influx of future IoT applications. The figure depicts a central IoT network hub, signifying the convergence point for multiple spectrum users. From the figure, incumbent users with longstanding rights to specific spectrum bands are shown. Adjacent to them are licensed or priority access users and GAA users. From a spectrum management perspective, spectrum sharing is coordinated by the database administrator. Incumbent users are afforded protection from harmful interference from all other lower priority users [31]. The priority access users are issued licences authorizing their use of the band with the condition that they may be requested by the SAS to modify their operating parameters to protect the incumbent users. Lastly, GAA users are afforded no protection and operate in a licence-exempt manner.

In general, the priority access users are cellular operators using the mid-band spectrum to augment their existing spectrum portfolio. However, some non-traditional auction winners are intending to implement private networks with potentially massive IoT networks [32]. From the perspective of the GAA users, this tier of user is expected to hold the greatest potential for IoT networks fuelled by a growing market of CBRS device capabilities [33,34,35]. Irrespective of the licensing approach for IoT services, the interconnections are mediated through an SAS to manage real-time spectrum allocation and interference.

Similar to the CBRS model, in the EU, the LSA approach, standardized by the European Telecommunications Standards Institute (ETSI), represents a two-tier system [36,37]. The LSA model is founded on a central geolocation database, which, unlike the CBRS system, typically holds static information about the incumbent users [4]. In addition, the LSA framework is progressively integrating environmental sensing capabilities to enable real-time analysis of atmospheric conditions. This ability not only has implications for identifying when the incumbent system is active, but is also a central component in dynamically tuning the spectrum in response to environmental variables, thereby increasing the access opportunities for IoT devices.

Additional instances of database-assisted sharing include TVWS and automated frequency coordination (AFC) [38,39,40]. The TVWS leverages unused segments within TV broadcast bands to support broadband devices within specific regions [41]. On the other hand, AFC manages spectrum use in the 6 GHz band, allocated for low and standard power usage in the US, by coordinating access through a database [42].

Irrespective of the sharing model—whether CBRS, LSA, TVWS, or AFC—the specific requirements for IoT deployments place additional constraints on database-assisted sharing. Generally, their need for flexible, immediate spectrum access highlights their requirements for real-time decision-making, that uses enhanced data sources. For instance, case studies such as TVWS in urban IoT networks [43], demonstrate how geospatial and environmental data improve spectrum sharing, showcasing benefits and practical challenges.

**Figure 2:**
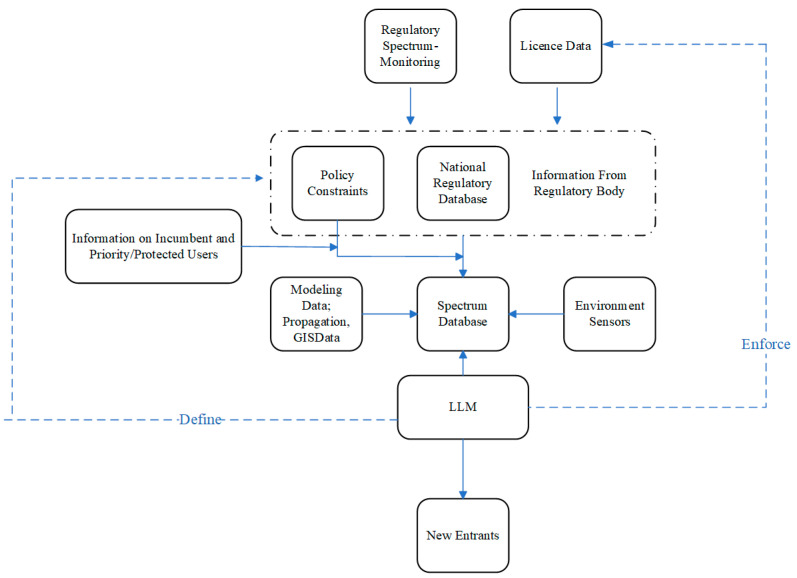
Block diagram of a revised spectrum database model including the flow of information in the system.

In the context of a revised general model for database-assisted spectrum sharing, Figure 2 depicts a functional block diagram of the enhanced sharing framework for IoT applications. In this case, a Large Language Model (LLM) is a critical component. The LLM serves as an intermediary between the new IoT entrants and the spectrum database. In this respect, the proposed system offers an enhancement over current state-of-the-art database-assisted systems such that it can harness advanced data from multiple sources, including incumbent and priority/protected user information, modelling data, regulatory constraints, and inputs from a national regulatory database and spectrum database.

The integration of high-resolution geospatial and real-time environmental data takes place within this enhanced system. To integrate high-resolution geospatial data, the system incorporates detailed terrain models, land use data, and clutter information, which provide a precise representation of the physical environment. These data are used to improve the accuracy of propagation models by considering the actual physical obstacles and variations in the terrain that affect signal propagation.

Real-time environmental data are integrated through environmental sensors deployed across the coverage area. These sensors continuously monitor atmospheric conditions, such as temperature, humidity, and precipitation, which can impact signal strength and interference levels. The data collected from these sensors are fed into the spectrum database, allowing the system to dynamically adjust spectrum allocations based on current environmental conditions.

Overall, the integration of this multifaceted data within the LLM, strengthens the decision-making framework, enabling it to process spectrum allocation requests with improved accuracy. The algorithms within the LLM, enhanced by machine learning and artificial intelligence (AI) methodologies, digest this complex dataset to support real-time spectrum sharing decisions. Furthermore, the LLM’s role extends to enforcing policy adherence through interactions with environment sensors and regulatory spectrum-monitoring systems, thus ensuring compliant and optimal usage of the spectrum. As new entrants seek access to the spectrum, the LLM evaluates their requests with respect to the current spectrum use, policy constraints, and real-time environmental factors.

## 5. Example Scenario: Sharing with Earth Stations in Mid-Band

An example scenario is presented, focused on incumbent satellite earth stations operating in mid-band spectrum in Canada. Specifically, the scenario examines and analyses the aggregate interference received by incumbent fixed satellite service (FSS) earth stations from stationary secondary services in the same geographical area. In this particular scenario, the secondary services include IoT sensors and mobile services. The frequency band under consideration, spanning from 3650 to 4200 MHz, resembles those used for CBRS; however, the approach and analysis is broadly applicable to the majority of bands identified for spectrum sharing. Within this specific band, there is a focus on incumbent services, particularly in areas adjacent to urban and suburban regions where spectrum demand tends to be higher compared to rural areas.

**Figure 3:**
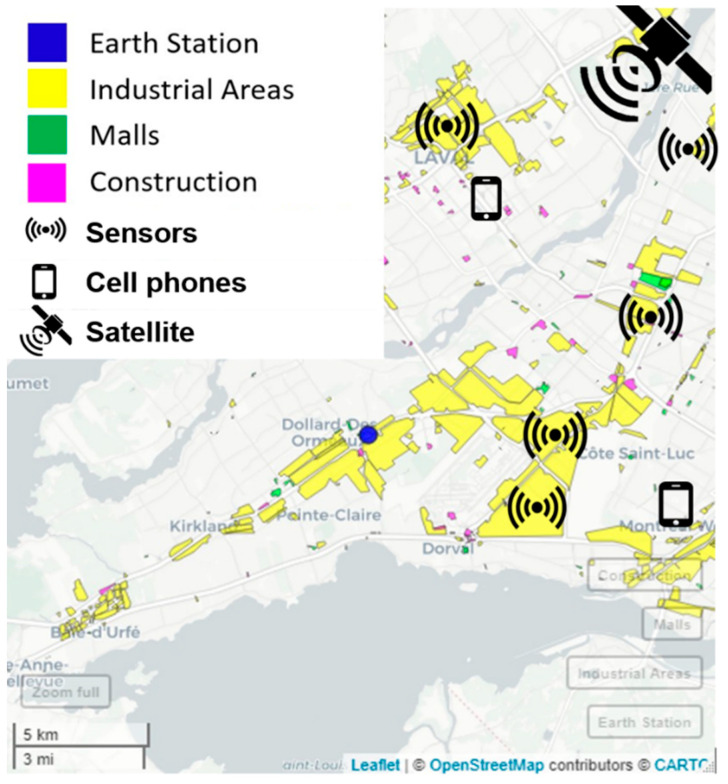
Map showing the location of the fixed satellite service earth station with geospatial footprint data taken from OpenStreetMap representing industrial areas, shopping malls and construction sites as locations for secondary systems.

For instance, Figure 3 illustrates the positioning of an incumbent satellite receiver earth station, as reported in the regulatory database [44], operating at 3925 MHz in Montreal, Canada. Technical specifications for this incumbent system are then extracted and listed in Table 1. Based on Figure 3 and in line with the evolving trend of spectrum sharing favouring more localized access, it is assumed that areas seeking secondary access to spectrum correspond to diverse vertical markets. In this scenario, the potential secondary transmitter locations are depicted through color-coded geospatial footprints, indicative of industrial zones, retail complexes, shopping centres, and construction sites. These geospatial footprints, sourced from OpenStreetMap [45], not only provide extra data for simulation analysis, but also serve as authentic representations of potential interference sources for the existing earth station.

We chose high-resolution geospatial data (CDEM and HRDEM) for accurate interference modelling, acknowledging increased computational complexity. OpenStreetMap data were used for realistic IoT deployment scenarios, though this selection may bias towards well-mapped areas [46].

Our simulation used high-resolution data from CDEM (0.75 arc seconds) and HRDEM (1 meter). We ran 3000 scenarios, placing potential secondary transmitters within OpenStreetMap geospatial footprints. We recorded I/N ratios, aggregate interference, and the number of accommodated transmitters under various conditions [47].

This data-driven approach contrasts with conventional methods, which typically assume a random distribution of potential secondary transmitters around the incumbent receiver, either homogeneously or population-weighted [48]. Moreover, it more closely reflects the demand patterns observed in vertical markets.

For the simulation analysis, the interference protection criteria (IPC) define the level at which the aggregate interference from all secondary services reduces the performance of the incumbent priority user to an unacceptable level. An IPC based on the interference-to-noise (I/N) ratio is commonly used to establish when harmful interference occurs. In the context of the CBRS system, a relatively conservative I/N threshold of −12 dB is applied to every FSS receiver station. Conversely, the AFC system sets a higher I/N threshold of −6 dB for licence-exempt devices.

Incorporating the revised parameters for IoT devices outlined in Table 2, the simulation adopts a data-driven approach to assess the IPC based on the I/N ratio. This methodology involves computing the aggregate interference at the output of the incumbent receiver, including the effects of the antenna gain pattern taken from the International Telecommunications Union–Recommendation (ITU-R) [49].

For the simulation, a set of $N_{c}$ geospatial footprints, sourced from OpenStreetMap, represents the potential locations of secondary IoT transmitters. These locations are determined as the centroids of the geospatial footprints, which denote the latitude and longitude of a single transmitter. The updated parameters of Table 2 reflect the typical features of IoT devices, such as their variable effective isotropic radiated power (EIRP), bandwidth, and antenna configurations. They account for the typical requirements of IoT applications, including indoor/outdoor antenna placement, high device density, and varied communication periodicity. Moreover, the secondary IoT systems are assumed to operate co-channel with incumbent systems, with path loss calculations derived from the geospatial footprints determining the mutual interference levels. In the scenario, the aggregate interference is calculated between the secondary systems and the incumbent as well as between the secondary systems themselves. Let $I_{i,j}$ denote the interference power at receiver *j* from transmitter i. The interference, in dBm, between two points is defined as follows:

(1) $$I_{i,j}=P_{i}+G_{i}+P_{i}(\theta_{i})-L_{p}(i,j)+G_{j}+P_{j}(\theta_{j})$$

where

$P_{i}$: Power (dBm) from the *i*th transmitter location

$G_{i}$,$G_{j}$: Antenna gain (dBi) for the *i*th transmitter and *j*th receiver, respectively

$P_{i}(\theta_{i})$,$P_{j}(\theta_{j})$: Correction gain (dB) based on the antenna gain pattern at an angle, $\theta_{x}$, between the *i*th transmitter and *j*th receiver, respectively

$L_{p}(i,j)$: Path loss (dB) between the *i*th transmitter and *j*th receiver

Assuming that there are $N_{a}$ simultaneously active secondary systems, where $N_{a}\leq N_{c}$, and J incumbent systems, and using the notation of $I_{i,j}^{mW}=10^{\left(I_{i,j}/10\right)}$, where $I_{i,j}^{mW}$ is the interference power in milliwatts (mW), the aggregate interference between secondary and incumbent is given by the following:

(2) $$I_{SI}=\sum_{i}^{N_{a}}I_{i,j}^{mW}$$

where *i* = 1, 2, …, $N_{a}$ and *j* = 1, 2, …, J. Similarly, between the secondary-to-secondary systems, the aggregate interference is calculated as follows:

(3) $$I_{SS,n}=\sum_{i}^{N_{a}}I_{i,n}^{mW}i\neq n$$

where *n* = 1, 2, …, $N_{a}$.

Within each simulation trial, a candidate secondary system location, from the predefined set, $N_{c}$, is selected from the geospatial footprints surrounding the incumbent system. The geospatial footprints represent realistic IoT device deployments in urban landscapes. For each candidate location, the aggregate interference is calculated between the candidate location, and the incumbent and any previously assigned secondary systems within the simulation. Depending on whether the I/N threshold has been exceeded or not, the system may be allocated a channel or denied access. The process is repeated until a scenario is reached whereby the collective aggregate interference at the incumbent breaches the I/N threshold or when all potential secondary IoT locations have been exhausted [50].

For the interference calculation, the precision of the point-to-point interference calculation is heavily reliant on the choice of propagation model. For the analysis, the following two models are employed: the Irregular Terrain Model (ITM) [51] and the ITU-R P.1812 [52].

**Figure 4:**
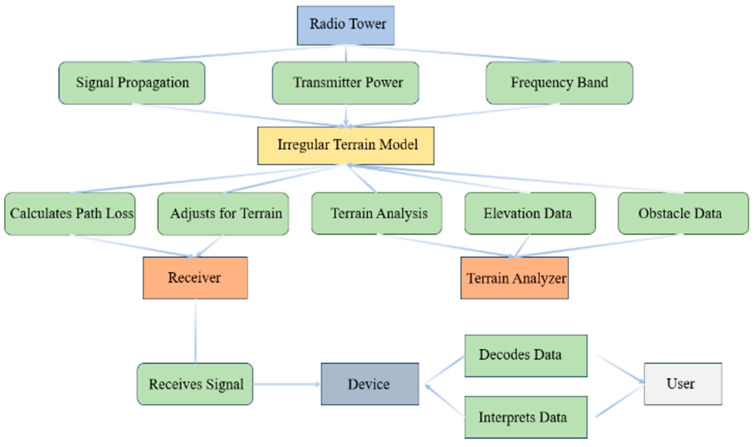
ITM propagation diagram.

The ITM, commonly referred to as the Longley-Rice model, is designed to predict electromagnetic wave propagation loss over irregular terrain for a wide range of frequencies, as shown in Figure 4. It considers the variability of terrain features, atmospheric conditions, and other environmental factors, making it suitable for assessing the potential interference in complex urban landscapes where IoT devices are typically deployed.

**Figure 5:**
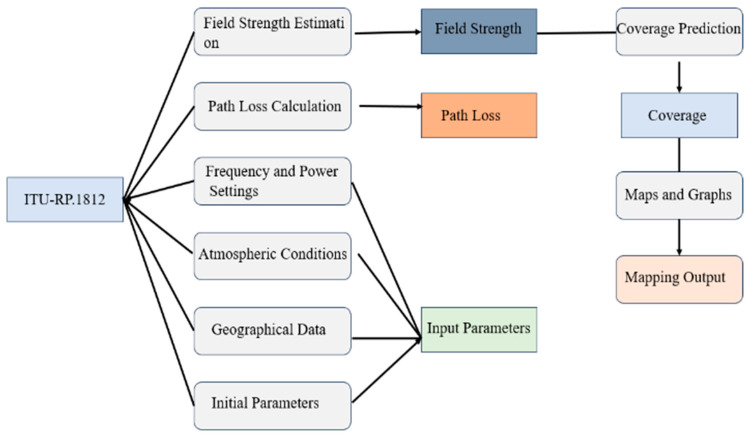
ITU-R P.1812 propagation prediction method diagram.

The ITU-R P.1812 model is a point-to-area propagation model as shown in Figure 5. It is optimized for predicting signal propagation in the VHF and UHF bands and is particularly adept at handling the influence of terrain undulations and urban structures on wave propagation over wide geographical areas. This model is often employed for planning broadcast services and for understanding how signals travel over diverse environments, including cityscapes where the incumbent and secondary systems are situated.

## 6. Simulation Results

We analysed data using histograms, CDFs, box plots, and line plots to compare different models and thresholds. We quantified improvements in spectrum sharing efficiency by calculating the percentage increase in accommodated secondary transmitters, using an 80% confidence level in CDF analysis.

Multiple analysis techniques were employed to provide comprehensive results and mitigate potential biases. Our study’s limitations include a focus on a single incumbent earth station and assumption of static IoT deployments, which may not fully capture real-world complexities.

**Figure 6:**
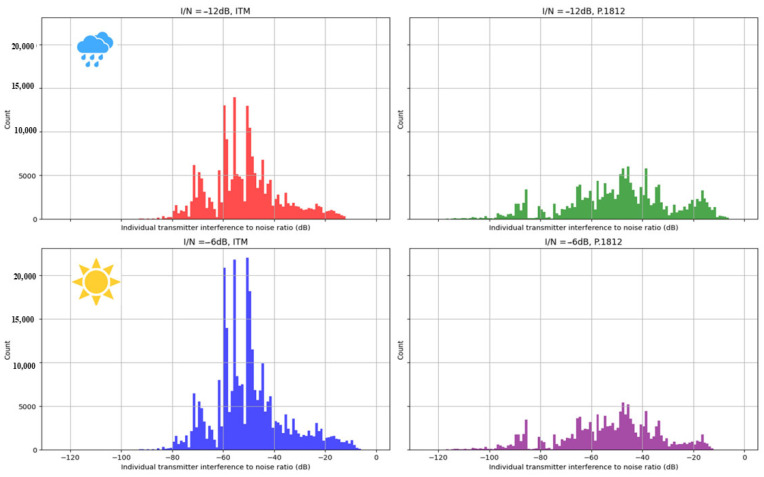
Histograms of the I/N ratio for individual (secondary) transmitters. Comparison of threshold values of −12 dB (**top** plots) and −6 dB (**bottom** plots) and the impact of propagation models ITM (**left**) and P.1812 (**right**).

Figure 6 provides an illustrative representation of the I/N histograms emanating from each individual secondary transmitter site that was active during our simulations. These sites were those that had been allocated a frequency channel and were authorized to transmit. The figure depicts the aggregation of I/N ratios from 3000 independent simulation scenarios. The aim is to show the consequences of adjusting the interference threshold, moving from a conservative −12 dB in the top histograms to a less stringent −6 dB in the bottom ones. In parallel, Figure 6 highlights the repercussions of leveraging extra datasets within our analysis. This comparative approach underscores two distinct propagation models incorporating divergent geospatial data inputs. The histograms broadly indicate a rise in the average number (count) of secondary transmitters permitted within the simulations when the interference threshold is augmented from −12 dB to −6 dB. This trend is consistent when contrasting the ITM and P.1812 models.

For the ITM, there is an integration of the Canadian Digital Elevation Model (CDEM) [53], which is instrumental in capturing the nuanced terrain interplay between the transmitter and receiver sites. The choice of ITM is far from arbitrary; it is a model widely regarded and employed by regulatory authorities, primarily for its efficacy in predicting signal coverage patterns as well as evaluating the potential for harmful interference in a given scenario.

In contrast, for the ITU-R P.1812 model, the CDEM data continues to serve as an input. However, it is the incorporation of the High-Resolution Digital Elevation Model (HRDEM) data [54] that marks a significant difference. This dataset is particularly relevant for propagation studies as it is capable of capturing both terrain contours and the ‘clutter’—buildings, trees, and other structures—that rises above ground level. The incorporation of such data into the P.1812 model promises path loss estimates that bear a closer resemblance to the multifaceted and cluttered environments that characterize modern propagation landscapes.

The data expressed in the histograms reveal the trend of an average increase in the count of secondary transmitters that are accommodated within the simulations. This shift becomes more pronounced when the interference threshold is elevated from −12 dB to −6 dB. This phenomenon does not emerge in isolation; it can be linked to the more conservative path loss attenuations introduced by the P.1812 model, which duly accounts for the additional above-ground clutter.

**Figure 7:**
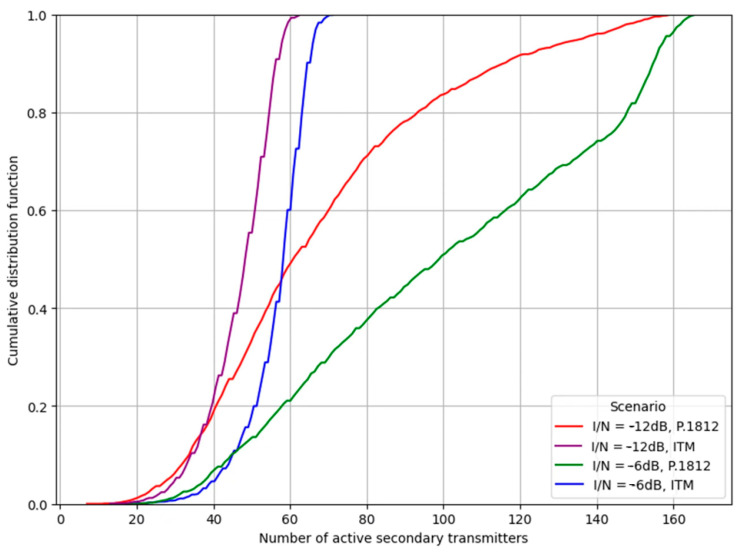
CDF of the I/N ratio for individual (secondary) transmitters. Comparison of threshold values of −12 dB and −6 dB and propagation models ITM and P.1812.

Histograms provide a visual measure of data spread and the presence of outliers. However, cumulative distribution functions (CDFs) allow the different results to be compared more readily in a single figure. Figure 7 charts the CDFs for each of the four combinations of interference thresholds and propagation models under review. A closer examination of the CDFs, particularly when the I/N ratio stands at −12 dB, reveals a tangible difference between the curves of ITM and P.1812 models. For simulation trials exceeding the 30% threshold, it becomes evident that secondary transmitters have a consistent and increased interference footprint. The inference here is fairly straightforward—as the P.1812 model adjusts, the path loss values upwards to reflect real-world attenuation. The change naturally allows for a larger array of secondary systems to transmit. This, in turn, culminates in an uptick in the mean I/N ratio.

At the −6 dB threshold, the separation between the curves becomes more pronounced, indicating a greater differentiation in performance between the ITM and P.1812 models. This observation can be ascribed to the broader selection pool of secondary locations now permissible under the −6 dB regime. This pool includes not only more distant sites but also those in close proximity or with unobstructed sightlines to the incumbent systems.

Specifically, the results show that when changing from −12 dB to −6 dB for the ITM model, 80% of the trials indicate an increase of approximately 17% in the number of secondary IoT users. For the P.1812 model, the increase is 60% when moving from −12 dB to −6 dB. Additionally, comparing the CDF curves between models at −12 dB, moving from ITM to P.1812 shows a gain of 77% in the number of new users supported at the 80% level. At −6 dB, the change from ITM to P.1812 results in a gain of 140%.

**Figure 8:**
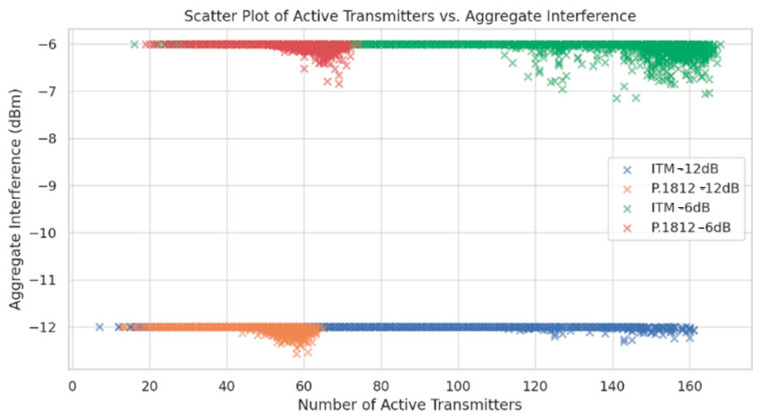
Relationship between transmitter density and aggregate interference levels.

Figure 8 provides insights into the relationship between the number of active transmitters and aggregate interference levels, revealing how different interference thresholds and propagation models influence spectrum sharing efficiency. The plot demonstrates a clear relationship between transmitter counts and aggregate interference levels. This relationship indicates how transmitter density directly impacts the interference levels in spectrum sharing scenarios, emphasizing the importance of balanced transmitter deployment to effectively manage interference.

The scatter plot also reveals the impact of different interference thresholds on aggregate interference levels. At a −12 dB interference threshold, aggregate interference levels remain relatively low across all transmitter counts, indicating that a conservative threshold effectively limits interference, even with higher transmitter densities. In contrast, at a −6 dB interference threshold, aggregate interference levels show a noticeable increase, suggesting that a higher threshold allows for more transmitters but at the cost of higher interference levels.

Furthermore, the scatter plot highlights differences between the ITM and P.1812 models. The ITM model allows for higher transmitter counts but exhibits greater fluctuations in interference levels, particularly at the −6 dB threshold. In comparison, the P.1812 model’s interference levels show less fluctuation, suggesting that its incorporation of advanced geospatial and environmental data provides a more stable interference environment.

These scatter plot insights reinforce the importance of managing transmitter counts and interference thresholds in spectrum sharing scenarios. The relationship between transmitter density and interference highlights the need for balanced deployment to maintain efficient spectrum sharing and manage interference levels. Additionally, the stable interference levels in the P.1812 model suggest that incorporating advanced geospatial and environmental data can create a more controlled spectrum sharing environment, supporting denser IoT deployments.

**Figure 9:**
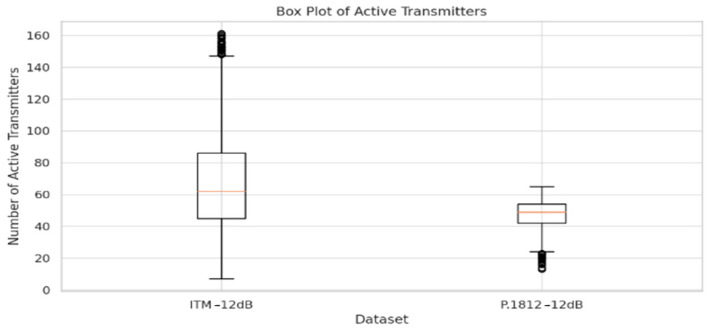
Box plot of active transmitters: ITM −12 dB vs. P.1812 −12 dB.

**Figure 10:**
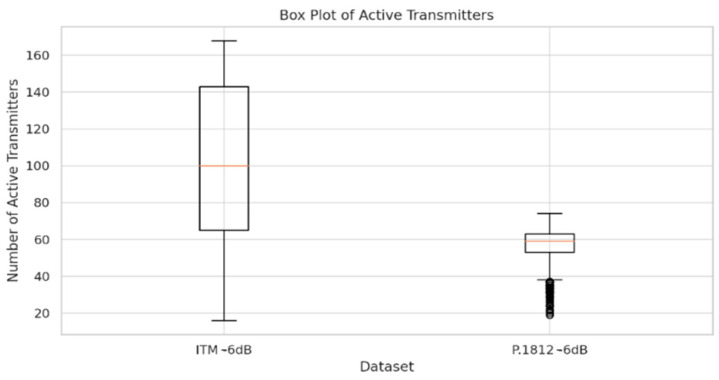
Box plot of active transmitters: ITM −6 dB vs. P.1812 −6 dB.

The box plots, as shown in Figure 9 and Figure 10, provide insights into the distribution of active transmitter counts across different datasets and configurations, shedding light on the impact of propagation models and interference thresholds on spectrum sharing efficiency.

The first box plot compares the distribution of active transmitters under the ITM −12 dB and P.1812 −12 dB models. This comparison highlights how the ITM model exhibits a wider range of active transmitters, indicating more variation in deployment. In contrast, the P.1812 model demonstrates a more concentrated distribution, suggesting a tighter clustering of transmitters. This difference in distribution reflects the characteristics of the two propagation models as follows: the ITM model’s wider range indicates a more dynamic transmitter deployment pattern, likely due to its sensitivity to terrain variations, allowing for greater diversity in transmitter locations. In contrast, the P.1812 model’s more concentrated distribution suggests that its incorporation of detailed environmental and geospatial data leads to a more controlled deployment pattern, reducing variation in transmitter counts.

The second box plot compares the distribution of active transmitters under a −6 dB interference threshold, showcasing the ITM and P.1812 models. In this configuration, the P.1812 model shows a lower median number of transmitters than the ITM model, indicating a more conservative deployment pattern. This result indicates how different interference thresholds and propagation models influence transmitter deployment. The P.1812 model’s lower median transmitter count reflects its more conservative approach, potentially due to its inclusion of detailed environmental data and its response to a −6 dB threshold, while the ITM model’s higher median count suggests that its sensitivity to terrain variations and interference thresholds allows for more dynamic transmitter deployment, even at a lower interference threshold.

These box plots illustrate how different propagation models and interference thresholds shape transmitter deployment patterns, influencing spectrum sharing efficiency and IoT deployments. The comparisons highlight how advanced geospatial and environmental data can guide transmitter distribution, resulting in more precise spectrum sharing frameworks. In summary, the data suggest that integrating these models and thresholds can support more efficient IoT deployments, allowing for better interference management and denser transmitter clustering, ultimately paving the way for more dynamic and adaptive spectrum sharing strategies.

**Figure 11:**
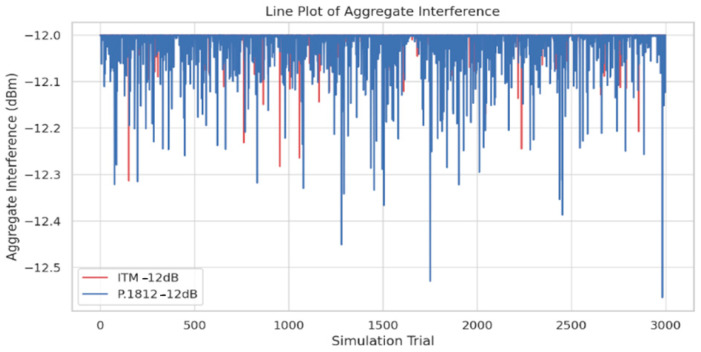
Line plot of aggregate interference: ITM −12 dB vs. P.1812 −12 dB.

**Figure 12:**
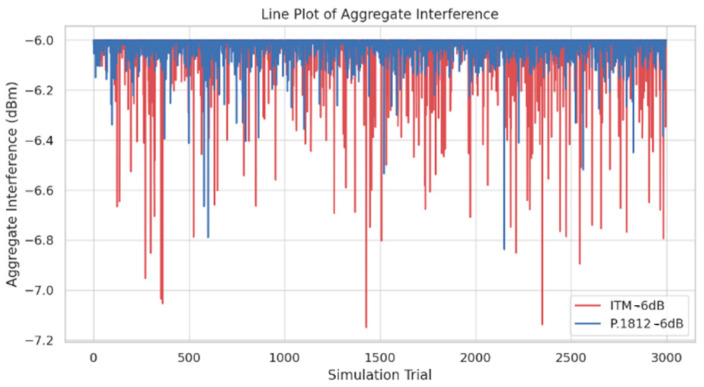
Line plot of aggregate interference: ITM −6 dB vs. P.1812 −6 dB.

The line plots in Figure 11 and Figure 12 provide insights for the variation of aggregate interference levels across different simulation trials, revealing how different propagation models and interference thresholds impact spectrum sharing efficiency. The findings from Figure 11 and Figure 12. reveal significant fluctuations in aggregate interference levels across trials, with the ITM model showing greater volatility compared to the P.1812 model. This variation can be attributed to the ITM model’s sensitivity to terrain variations and environmental factors, resulting in more variable propagation paths and interference patterns. In contrast, the P.1812 model, which incorporates advanced geospatial and environmental data, provides more stable interference levels by accounting for detailed environmental characteristics, including terrain contours and above-ground clutter.

Additionally, the figures illustrate the impact of different interference thresholds on aggregate interference levels. At a −12 dB threshold, interference levels remain relatively low across all transmitter counts, indicating that a conservative threshold effectively limits interference, even with higher densities. However, at a −6 dB threshold, there is a noticeable increase in aggregate interference levels, suggesting that a higher threshold allows for more transmitters but at the cost of increased interference. This highlights the need to choose appropriate propagation models and interference thresholds to balance transmitter deployment and interference management, impacting the efficiency of spectrum sharing frameworks.

## 7. Discussion

A detailed simulation of the sharing scenario was conducted, incorporating parameters from both the incumbent satellite earth station and secondary systems. The findings provide insights into the relationship between different interference thresholds, propagation models, and their impacts on spectrum sharing efficiency.

The analysis of simulation results reveals the following key insights:

1. The box plots of active transmitters show a distinct variation between the ITM and P.1812 models, particularly under different interference thresholds. At a −12 dB threshold, the ITM model exhibits a wider distribution range, indicating greater variation in deployment, while the P.1812 model shows a more concentrated distribution, reflecting its incorporation of advanced geospatial and environmental data. At a −6 dB threshold, the P.1812 model shows a lower median number of transmitters compared to the ITM model, indicating a more conservative deployment pattern.
2. The line plots of aggregate interference reveal differences in interference levels between the ITM and P.1812 models. The ITM model shows greater fluctuations, highlighting its sensitivity to terrain variations and other environmental factors, leading to more variable propagation paths and interference patterns. In contrast, the P.1812 model demonstrates more stable interference levels, reflecting its incorporation of advanced geospatial and environmental data, resulting in a more controlled spectrum sharing environment.
3. The scatter plot of interference levels illustrates the relationship between transmitter density and aggregate interference. At a −12 dB threshold, aggregate interference levels remain relatively low across all transmitter counts, indicating that a conservative threshold effectively limits interference, even with higher transmitter densities. In contrast, at a −6 dB threshold, there is a noticeable increase in aggregate interference levels, suggesting that a higher threshold allows for more transmitters, but at the cost of higher interference levels.
4. Comparison of Models: The comparison between the ITM and P.1812 models indicates that the ITM model allows for higher transmitter counts, but with greater fluctuations in interference levels, while the P.1812 model’s interference levels show less fluctuation.

Our simulation aims to evaluate the efficacy of an enhanced spectrum sharing framework that integrates high-resolution geospatial and real-time environmental data. Traditional spectrum sharing methods often rely on static database systems, which can be limited in their ability to adapt to the dynamic nature of modern IoT applications. While state-of-the-art technologies such as LSA in the European Union and SAS for the CBRS in the United States have introduced dynamic elements into spectrum management, they still predominantly depend on historical data and lack the granularity and responsiveness required for modern IoT environments.

For instance, LSA and SAS frameworks incorporate geolocation databases to facilitate spectrum sharing among primary users, secondary licensees, and general authorized access users. These systems, although pioneering, are inherently limited by their reliance on static data and their inability to incorporate real-time environmental variations, leading to suboptimal spectrum allocation and increased interference.

By modelling realistic deployment scenarios and using advanced propagation models such as the ITM and the ITU-R P.1812, our simulation provides a robust analysis of how our proposed framework can enhance spectrum sharing efficiency. The simulation results highlight the potential for increased access opportunities for IoT deployments and improved spectrum utilization, demonstrating that our approach can complement and extend the capabilities of existing state-of-the-art technologies.

Comparing our framework with state-of-the-art technologies, our results indicate that incorporating real-time environmental data and high-resolution geospatial information significantly improves the predictive accuracy and adaptability of spectrum management systems. This advancement is crucial for addressing the unique demands of IoT networks, which require dynamic and immediate spectrum access to function efficiently.

These simulation findings emphasize the importance of incorporating advanced geospatial and environmental data into spectrum sharing frameworks, allowing for more efficient and flexible IoT deployments. The integration of these data sources not only supports denser IoT deployments but also reduces interference and fosters dynamic spectrum sharing frameworks, accommodating the complex operational characteristics of IoT networks.

## 8. Comparative Analysis

Table 3 demonstrates that our LLM-enabled dynamic sharing approach consistently outperforms fixed sharing in terms of stability and efficiency across different weather conditions.

The LLM continuously analyses local geographical features and current meteorological data to predict short-term weather changes, enabling proactive adjustments to interference thresholds and spectrum allocation. This adaptive approach ensures efficient spectrum utilization and improved network stability across varying conditions. While it requires more sophisticated infrastructure, the benefits in terms of spectrum efficiency and network resilience are significant. These findings underscore the potential of our proposed method in enhancing spectrum sharing efficiency for IoT applications, particularly in environments with variable conditions and diverse geographical features, showcasing the transformative power of integrating advanced AI into spectrum management.

## 9. Conclusions and Further Work

This research highlights significant advancements in spectrum sharing by integrating advanced geospatial and environmental data into database-assisted systems, particularly for the mid-band spectrum. This integration enhances spectrum management by addressing the specific needs of vertical markets and IoT ecosystems.

With the inclusion of detailed geospatial datasets, including terrain models and clutter data, the study introduces more authentic interference models for both incumbent and secondary systems. This approach supports more dynamic spectrum sharing and reduces the reliance on worst-case scenario assumptions, leading to improved spectrum availability for secondary IoT users. The integration of advanced data sources allows for more precise models of interference, particularly at FSS earth stations. This refinement facilitates a more balanced approach to spectrum sharing, where secondary users can access the spectrum without causing harmful interference to incumbent systems.

The research demonstrates gains in the number of new entrants accessing the spectrum, ranging from 77% to 140%, when moving to less conservative interference conditions and including more complex geospatial information on the propagation environment. Incorporating advanced environmental data and high-resolution geospatial datasets, such as the CDEM and HRDEM, enhances the accuracy of propagation models such as ITM and ITU-R P.1812. These models, in turn, contribute to a more controlled spectrum-sharing environment, supporting denser IoT deployments. The study also emphasizes the importance of accommodating the diverse needs of IoT networks, particularly in industrial and smart city settings. These scenarios require flexible, real-time spectrum allocation mechanisms that leverage detailed data sources, enabling more efficient IoT operations and reducing interference.

Future research will examine various IoT network scenarios to refine spectrum sharing strategies further. Adjustments to the IPC thresholds may be necessary to cater to the distinct operational characteristics of different IoT applications. The research also recommends incorporating a wider range of data sources into spectrum sharing frameworks, including advanced environmental sensing and nuanced IoT demand patterns. Ensuring the quality and reliability of these datasets is crucial for precise spectrum allocation.

The findings underscore the importance of integrating geospatial and environmental data into spectrum sharing frameworks, paving the way for more adaptive and dynamic spectrum sharing strategies. This integration not only supports denser IoT deployments but also fosters efficient spectrum management, catering to the complex demands of modern IoT ecosystems. Continued research and technological advancements will help sustain this progress, ensuring the growth and functionality of future digital ecosystems.

## Figures and Tables

**Table 1:** Parameters for the incumbent system in the simulation environment.

Parameter	Value
Antenna Height	10 m
Antenna Elevation Angle Degrees	36.8
Antenna Azimuth Angle	192.8 degrees
Antenna Pattern	ITU REC-465
Bandwidth	2 MHz
Noise Temperature	100 Kelvin
Frequency	3925.625 MHz
Latitude	45.48306 degrees
Longitude	−73.78806 degrees
Satellite ITU Name	USASAT-24V

**Table 2:** Parameters for the secondary systems in the simulation environment.

Parameter	Value
Transmitter EIRP	0–20 dBm
Bandwidth	1–5 MHz
Antenna Height	1–3 m (typical for loT devices)
Antenna Directionality	Directional (as needed for loT)
Antenna Gain	0–5 dBi
Downlink Traffic	Variable
Antenna Location	Indoor/Outdoor
Device Density	High
Communication Periodicity	Frequent or Infrequent
Parameter	Value

**Table 3:** Dynamic vs. Fixed Spectrum Sharing.

Method. (Spectrum Sharing)	Weather Condition	Interference Threshold (dB)	Number of Secondary Users	Network Stability	Gain
Fixed	Good (Sunny)	−12 dB	102,023	Moderate	Moderate
Fixed	Bad (Rainy)	−12 dB	102,023	Low	Low
Dynamic	Good (Sunny)	−6 dB	110,342	High	High
Dynamic	Bad (Rainy)	−12 dB	94,073	High	High

## Data Availability

Data are contained within the article.
